# Comparative study of electrocardiographic parameters in calves born after eutocia versus dystocia

**DOI:** 10.14202/vetworld.2022.2603-2610

**Published:** 2022-11-18

**Authors:** Julia Nowak, Jessica Joerling, Marlene Sickinger, Axel Wehrend

**Affiliations:** 1 Clinic for Veterinary Obstetrics, Gynaecology, and Andrology for Large and Small Animals with Ambulance Service, Faculty of Veterinary Medicine, Justus-Liebig-University Gießen, Frankfurter Straße 106, 35392 Giessen, Germany; 2 Clinic for Ruminants, Justus-Liebig-University Gießen, Frankfurter Straße 104, 35392 Gießen, Germany

**Keywords:** birth, bovine, electrocardiogram, neonate

## Abstract

**Background and Aim:**

The mortality rate of perinatal calves is high, particularly in dystocia cases. Besides detectable conditions such as trauma or amniotic fluid aspiration, the potential salience of cardiological diseases in neonatal bovine deaths has received little attention. This study aimed to compare the electrocardiographic parameters of calves born under conditions of dystocia and eutocia.

**Materials and Methods:**

Electrocardiographic, clinical, and laboratory diagnostic examinations were performed during the first 5 days of life on 40 calves. Of them, 20 calves were born under conditions of dystocia and 20 of eutocia.

**Results:**

Electrocardiograms (ECGs) did not show detectable arrhythmias in all calves. Both groups exhibited tachycardia on their first ECGs. The QT and ST interval durations developed differently over time in both groups, suggesting that these may be related to conditions of birth.

**Conclusion:**

The electrocardiographic differences between calves born of dystocia and eutocia could be a factor in the increased mortality rate of calves born of dystocia.

## Introduction

The perinatal period is crucial in the survival of calves [1]. As approximately 90% of calves that die perinatally are still alive at the beginning of calving, birth management is essential [1]. Perinatal mortality in dairy farms is 8.5% in the Netherlands and 2.0% in Norway [2, 3], while in suckler farms, 5.1% in the United Kingdom [4]. Calf mortality within the first 24 h of life on German dairy farms (Holstein Friesian and Black Pied Dairy Cow) is 9.3% compared to other countries [5]. Dystocia has been significantly associated with increased perinatal mortality (p < 0.001). A Swedish study examined calves that died perinatally: Half of them were born of dystocia, while 5% died from congenital defects, and one-third of the calves died without any apparent disease [6]. The causes of increased mortality in calves after dystocia include trauma (fractures and internal bleeding), anoxia (pulmonary atelectasis, meconium aspiration, and collapsed trachea), infections, and surfactant deficiency [1, 4]. However, some deaths have unexplained causes.

In bovine neonates, electrocardiograms (ECGs) have been used to compare the cardiac performance of different breeds of cattle [7] and to study the effects of different drugs on cardiac activity [8, 9]. Among the primary causes of death are arrhythmias due to electrolyte imbalances based on the electrical excitation of the myocardium of calves suffering from neonatal diarrhea [10]. The ECGs are more commonly used in calves to diagnose viral myocarditis and to monitor and treat resulting arrhythmias [11, 12]. The ECG also plays an increasingly important role in further diagnostics [13].

An ECG can be performed on a long-term or short-term basis, depending on the presenting issue [14, 15]. For the latter, smartphone-compatible software can be compatible to the standard ECG, thus allowing its use in the field [16].

This study aimed to investigate whether electrocardiographic parameters differ in calves born under conditions of dystocia and eutocia using an ambulatory ECG to improve postnatal health monitoring and, if necessary, treatment.

## Materials and Methods

### Ethical approval and informed consent

Examinations and treatments were performed according to the standard therapeutic measures without any unnecessary harm to the animals. A signed consent was obtained from each patient’s owner before the study.

### Study period and location

The study was conducted from February 2014 to May 2015 in the Clinic for Veterinary Obstetrics, Gynecology and Andrology for Large and Small Animals in Gießen and surrounding cattle farms in the Hessen region (central Germany).

### Technical equipment

**Figure-1:**
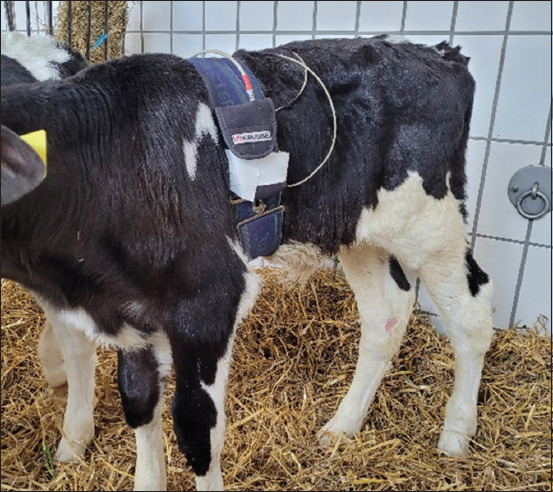
Healthy Holstein Friesian calf during the recording of the electrocardiogram.

The electrocardiographic measurements were assessed using the battery-powered, telemetrically operating Televet 100 device (Engel Engineering Service GmbH, Heusenstamm, Germany), patient cables, and self-adhesive disposable electrodes (SilverTRACE^®^ ECG Electrodes Soft P55MO, Leonard Lang GmbH, Innsbruck, Austria) (Figure-1). Data were transmitted telemetrically through Bluetooth to a tablet equipped with Televet 100 software, version 4.1.3 (Engel Engineering Service GmbH).

### Blood sample collection and laboratory diagnostic determination

Blood samples were collected from the jugular vein within 24 h postpartum. Hematocrit, pH, base excess, and concentrations of sodium, potassium, ionized calcium, glucose, and lactate were measured using a blood gas analyzer (ABL 800 Basic Analyzer, Radiometer, Copenhagen, Denmark) (Table-1). In Group 1, the collection of a venous blood sample followed the clinical and electrocardiographic examination. In Group 2, venous blood sampling was delayed by initial postnatal resuscitation measures.

**Table-1:** Group comparison of laboratory diagnostic measurements of calves (n = 40) from eutocia and dystocia during the first 24 h postpartum for variables of pH, ABE, Hct, Na^+^, K^+^, Ca^2+^, Glu, and Lac.

Parameter	Reference range	Eutocia	Dystocia	p-value
pH	7.36–7.46	7.39 ± 0.05 (7.3–7.54)	7.30 ± 0.07 (7.16–7.4)	<0.0001
ABE (mmol/l)	−0.6–4.6	5.2 ± 2.0 (1.8–9.7)	1.7 ± 4.8 (−6.6–1.3)	0.0056
Hct (%)	28–38	32 ± 7 (22–46)	33 ± 5 (26–41)	0.7936
Na^+^ (mmol/l)	130–150	136 ± 2 (131–139)	137 ± 3 (132–143)	0.1224
K^+^ (mmol/l)	3.5–5.5	4.5 ± 0.4 (3.8–5.5)	4.2 ± 0.4 (3.4–5.0)	0.0544
Ca^2+^ (mmol/l)	1.12–1.30	1.27 ± 0.06 (1.17–1.38)	1.34 ± 0.05 (1.26–1.48)	0.0004
Glu (mmol/l)	4.9–6.2	5.1 ± 1.6 (2.0–9.5)	5.2 ± 2.3 (2.5–11.7)	0.7964
Lac (mmol/l)	0.9–4.4	3.2 ± 1.3 (1.1–6.4)	6.5 ± 3.0 (2.1–12.5)	0.0001

ABE=Base excess, Hct=Hematocrit, Na^+^ =Sodium, K^+^ =Potassium, Ca^2+^ =Ionized calcium, Glu=Glucose, Lac=Lactate

### Calf grouping

A total of 40 calves were included and divided into two groups of 20. Grouping was not based on criteria of sex, breed, housing type, or feeding management.

Group 1 comprised calves not older than 24 h and were born spontaneously or easily by obstetric care. The inclusion criteria for this group were that calves must have been carried to term and had no abnormalities in the clinical examination on the 1^st^ day of life. Calves placed in this group had ECGs taken for 5 consecutive days.

Group 2 comprised calves delivered (either by cesarean section or a difficult extraction) by cows presenting with dystocia at the Clinic for Obstetrics, Gynecology, and Andrology of Large and Small Animals with Ambulatory Service of the Justus-Liebig-University in Gießen. The inclusion criteria included being carried to term, the absence of disease in the calves during the study period, and at least two consecutive ECGs.

### Investigations

All calves underwent general clinical and segmental examinations within 24 h postpartum, before ECG examination for group classification. During the following 4 days, ECG examinations were performed before clinical examinations to control any potential influence of the ECG. For the ECG examination, an elastic belt with the Televet 100 device was attached and fastened around the circumference of the thorax, directly behind the shoulder limbs. Lead II, a base apex lead, was used for evaluation. Each measurement lasted 15 min. The electrodes were left on the calf’s body over the 5-day assessment to prevent deviations in the ECG caused by alterations in electrode placement. A heart rate of 120–150 beats per minute was the reference value for a neonate’s heart rate [17].

Group 1: In the healthy calves, the first ECG was recorded as soon as possible after birth, followed by the collection of a venous blood sample. Further measurements were taken during the next 4 days at 24 h intervals to avoid ECG deviations due to diurnal variation. To prevent stress-related ECG artifacts, the animals were left in their usual environment without restraints, and no measurements were taken directly before or during feeding.

Group 2: In the dystocia group, all measures necessary for resuscitation (suction of amniotic fluid from the mouth and nose, stimulation of circulation by massaging the thorax, injection with doxapram if spontaneous respiration failed, umbilical care, and/or administration of oxygen) and first aid were taken immediately after birth. The first ECG was recorded and a venous blood sample was taken only when calves were no longer in a life-threatening situation and a segmental clinical examination had been performed. The subsequent measurements took place on the following 4 days at intervals of 24 h. As the calves were healthy and did not require further treatment in the clinic, eight calves in this group were discharged before the 5^th^ day postpartum. Therefore, all calves with at least two ECG measurements remained in the group.

### Statistical analysis

Statistical analyses were conducted using the statistical program package BMDP/Dynamic, Release 8.1 (Statistical Solutions Ltd.; Ireland). When analyzing group comparisons, a distinction was made between measures that were collected only once versus process data. Singular measures included the comparison of laboratory diagnostic values, as these were determined only on the 1^st^ day of life in both groups. Between-group analysis was conducted using the Wilcoxon-Mann-Whitney test. Furthermore, correlation analyses were carried out on laboratory diagnostic parameters, vital signs, and the electrocardiographic measurements of the 1^st^ day of life.

Follow-up data included ECG parameters (P wave duration, QRS complex duration, T wave duration, PQ interval duration, QT interval duration, and ST interval duration) as well as vital signs (heart rate, respiratory rate, and body temperature). A two-factor, repeated measures analysis of variance was conducted, with the help of the Wald test for missing values. Significant changes in variables in relation to time, group membership, and their interactions were examined.

## Results

A total of 191 ECGs from 40 neonatal calves were available for evaluation. Eight calves belonging to the dystocia group were discharged after the second ECG was taken.

Group 1: All neonates born spontaneously or with the help of basic obstetric care were deemed healthy (Table-2). The laboratory diagnostic results are summarized in Table-1. Values for the duration of P waves, QRS complexes, T waves, PQ intervals, QT intervals, and ST intervals are shown in Table-3. All calves in Group 1 demonstrated steady and regular cardiac action in all ECGs. Five calves exhibited tachycardia in the first measurement (162–194 beats per minute). No arrhythmias occurred.

**Table-2:** HR, RR, and internal body temperature in °C of bovine neonates (n days 1–3 = 40, n day 4 = 39, and n day 5 = 32) from eutocia and dystocia during the first 5 days of life.

Parameter	Eutocia	Dystocia	Eutocia	Dystocia	Eutocia	Dystocia	Eutocia	Dystocia	Eutocia	Dystocia
				
	Day 1 p. n.		Day 2 p. n.		Day 3 p. n.		Day 4 p. n.		Day 5 p. n.	
HR (min–max)	172 ± 16 (142–194)	173 ± 16 (150–200)	149 ± 18 (114–179)	155 ± 20 (120–188)	138 ± 16 (108–165)	147 ± 13 (123–170)	123 ± 18 (98–157)	143 ± 12 (118–169)	108 ± 15 (73–137)	133 ± 15 (102–156)
RR (min–max)	60 ± 4 (52–68)	59 ± 4 (52–68)	54 ± 5 (48–64)	53 ± 4 (48–64)	50 ± 6 (44–60)	49 ± 4 (40–56)	45 ± 5 (40–60)	47 ± 3 (44–52)	41 ± 5 (36–52)	44 ± 4 (36–48)
Temp (min–max)	39.1 ± 0.2 (38.7–39.4)	39.1 ± 0.2 (38.9–39.4)	38.8 ± 0.2 (38.5–39.1)	38.9 ± 0.2 (38.7–39.4)	38.7 ± 0.1 (38.4–38.9)	38.7 ± 0.1 (38.5–39.0)	38.5 ± 0.1 (38.4–38.8)	38.6 ± 0.1 (38.4–38.9)	38.5 ± 0.1 (38.3–38.8)	38.5 ± 0.1 (38.4–38.7)

HR=Heart rate, RR=Respiratory rate, Temp=Temperature in °C

**Table-3:** Measures in milliseconds for the duration of ECG parameters of P wave, QRS complex, T wave, QT interval, PQ interval, and ST interval and the mean HR of electrocardiograms of bovine neonates (n days 1–3 = 40, n day 4 = 39, and n day 5 = 32) from eutocia and dystocia, during the first 5 days of life.

Parameter	Eutocia	Dystocia	Eutocia	Dystocia	Eutocia	Dystocia	Eutocia	Dystocia	Eutocia	Dystocia
				
	Day 1 p. n.		Day 2 p. n.		Day 3 p. n.		Day 4 p. n.		Day 5 p. n.	
P wave	72 ± 10	57 ± 6	71 ± 9	59 ± 7	73 ± 9	62 ± 8	73 ± 7	60 ± 6	76 ± 9	62 ± 6
QRS complex	53 ± 7	47 ± 6	55 ± 7	48 ± 5	57 ± 7	49 ± 6	57 ± 7	48 ± 5	57 ± 6	47 ± 4
T wave	83 ± 8	78 ± 10	88 ± 9	82 ± 10	94 ± 9	85 ± 9	99 ± 12	86 ± 11	100 ± 9	92 ± 10
QT interval	212 ± 19	212 ± 12	232 ± 21	229 ± 18	239 ± 19	234 ± 13	256 ± 20	237 ± 13	270 ± 18	245 ± 15
PQ interval	118 ± 14	113 ± 13	120 ± 13	113 ± 12	122 ± 13	115 ± 16	129 ± 13	115 ± 12	133 ± 16	119 ± 15
ST interval	75 ± 18	87 ± 14	88 ± 18	99 ± 19	88 ± 21	100 ± 15	100 ± 17	102 ± 13	113 ± 16	106 ± 15
HR	172 ± 16	173 ± 16	149 ± 18	155 ± 20	138 ± 16	147 ± 13	123 ± 18	143 ± 12	108 ± 15	133 ± 15

HR=Heart rate, ECG=Electrocardiogram

Group 2: Of the 20 neonates from the dystocia group, nine exhibited stable vital signs immediately after birth, while the remaining 11 demonstrated depressed vital signs, which primarily manifested as respiratory disturbances. Data pertaining to vital signs, laboratory diagnostic measurements, and results of the ECGs of the calves from complicated births are shown in Tables-1–3. Nine calves exhibited tachycardia at the first measurement (163–200 beats per minute). All calves in this group demonstrated even and regular heart activity in all ECGs and did not display arrhythmias. Over the entire duration of the study, there was a tendency for all ECG measurements (in milliseconds) to lengthen from the 1^st^ to the 5^th^ day of life (Table-3).

There were significant between-groups differences in the laboratory diagnostic values for pH and base excess. Calves born of dystocia had significantly lower pH values (p < 0.005) and significantly higher ionized calcium and lactate concentrations (p < 0.0004) in their blood (Table-1).

The two-factor analysis of variance demonstrated a significant change in all ECG measures over time (p < 0.0001; Table-4). All ECG parameters, except ST interval duration, were significantly different between groups (p < 0.01). Significant interactions (p < 0.05) between group and time were only present for QT interval duration and ST interval duration. Correlation analyses were performed to investigate possible correlations between laboratory diagnostic values and ECG parameters or vital signs. These analyses were carried out per group, as the laboratory diagnostic measurements and ECG and vital signs measures differed significantly between the groups. In the correlation analysis of calves from spontaneous deliveries (Table-5), there was a significant difference between P wave duration and pH, P wave duration and heart rate, QT interval duration and heart rate, ST interval duration and heart rate, and for T wave duration and internal body temperature (p ≤ 0.05). The heart rate was positively correlated to P wave duration, while the other measures were negatively correlated with each other. The strongest linear correlation (r = −0.75) was found between heart rate and ST interval duration. Correlation analyses of calves born of dystocia (Table-6) showed a significant difference between QT interval duration and potassium concentration, QT interval duration and lactate concentration, heart rate and QT interval duration, heart rate and ST interval duration, and respiratory rate and QT interval duration (p ≤ 0.05). A positive relationship was found between potassium concentration and QT interval duration, while the remaining variables demonstrated inverse relationships with each other. The strongest correlation was found between heart rate and QT interval duration (r = −0.84).

**Table-4:** Results of the two-factor, repeated measures analysis of variance, regarding the time, using the Wald test for the progression data PDu, QRSDu, TDu, QTDu, PQDu, STDu, HR, RR, and internal body temperature to compare the groups (eutocia and dystocia) and observation time points.

Variable	Effects (p-Wert)		Interaction (p-Wert) group x time

	Group	Time	
PDu	<0.0001	<0.0001	0.1887
QRSDu	<0.0001	<0.00191	0.1845
TDu	0.0001	<0.0001	0.2730
QTDu	0.0062	<0.0001	0.0003
PQDu	0.0060	<0.0001	0.2462
STDu	0.1465	<0.0001	0.037
HR	0.0017	<0.0001	0.0004
RR	0.9357	<0.0001	0.0247
Temp	0.6649	<0.0001	0.4523

PDu=P wave duration, QRSDu=QRS complex duration, TDu=T waves, QTDu=QT interval, PQDu=PQ interval, STDu=ST distance, HR=Heart rate, RR=Respiratory rate, Temp=Temperature

**Table-5:** Results of the correlation analysis of the electrocardiographic measurements of PDu, QRSDu, TDu, PQDu, QTDu, and STDu; and vital signs of HR, RR, and internal body temperature in relation to laboratory diagnostic values for variables of pH, ABE, Hct, Na^+^, K^+^, Ca^2^+, Glu, and Lac and vital parameters of calves born from eutocia on the 1^st^ day postpartum, with correlation coefficient/p-value.

Parameter	pH	ABE	Hct	Na^+^	K^+^	Ca^2+^	Glu	Lac	HR	RR	Temp
PDu	−0.469/0.037	0.042/0.859	0.135/0.570	−0.209/0.378	−0.161/0.498	0.151/0.525	−0.130/0.586	0.127/0.595	0.508/0.022	0.102/0.670	−0.281/0.231
QRSDu	0.174/0.462	0.258/0.252	0.127/0.594	−0.300/0.198	−0.191/0.421	−0.091/0.701	0.150/0.528	−0.276/0.238	0.133/0.575	0.006/0.980	0.050/0.834
TDu	−0.407/0.075	−0.164/0.489	0.214/0.364	−0.266/0.257	−0.22/0.925	0.065/0.785	−0.130/0.584	0.192/0.416	0.389/0.090	−0.092/0.700	−0.485/0.030
PQDu	−0.191/0.421	0.132/0.578	0.193/0.415	0.231/0.326	0291/0.354	−0.314/0.177	−0.333/0.152	0.392/0.088	−0.201/0.396	−0.224/0.343	−0.152/0.523
QTDu	0.116/0.626	0.142/0.549	0.125/0.600	−0.210/0.374	0.143/0.546	−0.127/0.594	0.176/0.457	−0.155/0.630	−0.497/0.026	−0.176/0.458	−0.021/0.930
STDu	0.245/0.298	0.129/0.588	−0.016/0.946	0.016/0.948	0.230/0.328	−0.128/0.589	0.189/0.426	−0.106/0.656	−0.747/<0.001	−0.143/0.548	0.184/0.437
HR	−0.056/0.816	−0.041/0.863	0.146/0.538	−0.317/0.173	−0.156/0.512	0.123/0.606	0.090/0.706	0.026/0.913			
RR	−0.104/0.663	0.184/0.437	−0.210/0.373	−0.148/0.533	0.271/0.247	0.365/0.114	0.451/0.046	−0.086/0.719			
Temp	0.258/0.272	0.332/0.153	−0.543/0.013	0.262/0.265	−0.339/0.144	0.087/0.717	0.200/0.398	−0.404/0.077			

PDu=P wave duration, QRSDu=QRS complex duration, TDu=T wave duration, PQDu=PQ interval, QTDu=QT interval, STDu=ST interval, HR=Heart rate, RR=Respiratory rate, Temp=Temperature, ABE=Base excess, Hct=Hematocrit, Na^+^=Sodium, K^+^=Potassium, Ca^2+^=Ionized calcium, Glu=Glucose, Lac=Lactate

**Table-6:** Results of the correlation analysis of the electrocardiographic measurements of PDu, QRSDu, TDu, PQDu, QTDu, and STDu; and vital signs of HR, RR, and internal body temperature in relation to the laboratory diagnostic values for variables of pH, ABE, Hct, Na^+^, K^+^, Ca^2+^, Glu, and Lac and the vital parameters of calves born from dystocia, on the 1^st^ day postpartum, with correlation coefficient/p-value.

Parameter	pH	ABE	Hct	Na^+^	K^+^	Ca^2+^	Glu	Lac	HR	RR	Temp
PDu	−0.213/0.368	−0.105/0.658	−0.062/0.794	−0.048/0.840	−0.056/0.814	−0.147/0.536	0.051/0.831	0.111/0.641	−0.148/0.533	0.187/0.430	0.010/0.967
QRSDu	0.102/0.669	0.117/0.624	−0.120/0.616	0.010/0.965	0.091/0.704	−0.200/0.397	−0.148/0.535	−0.270/0.251	−0.318/0.172	−0.059/0.805	0.114/0.631
TDu	−0.136/0.568	0.018/0.940	−0.072/0.763	−0.386/0.093	0.139/0.560	−0.348/0.133	0.254/0.279	0.075/0.754	0.036/0.880	0.041/0.865	−0.129/0.589
PQDu	0.237/0.315	0.076/0.751	0.049/0.838	0.119/0.618	−0.163/0.491	−0.060/0.802	0.004/0.987	−0.131/0.582	−0.402/0.079	−0.337/0.146	0.260/0.268
QTDu	0.203/0.390	0.228/0.333	−0.266/0.257	0.064/0.789	0.464/0.040	0.018/0.914	−0.209/0.377	−0.467/0.038	−0.840/<0.001	−0.532/0.016	−0.080/0.738
STDu	0.221/0.350	0.129/0.587	−0.122/0.608	0.314/0.77	0.249/0.291	0.331/0.154	−0.289/0.216	−0.330/0.155	−0.589/0.006	−0.440/0.052	−0.021/0.931
HR	−0.348/0.132	−0.273/0.244	0.198/0.404	−0.041/0.865	−0.384/0.094	0.154/0.156	0.100/0.675	0.574/0.008			
RR	−0.610/0.004	−0.448/0.048	0.165/0.486	0.160/0.501	−0.319/0.170	0.081/0.733	0.038/0.875	0.491/0.028			
Temp	−0.055/0.819	0.064/0.788	−0.005/0.983	0.149/0.529	−0.318/0.172	0.149/0.532	−0.015/0.950	0.094/0.692			

PDu=P wave duration, QRSDu=QRS complex duration, TDu=T wave duration, PQDu=PQ interval, QTDu=QT interval, STDu=ST interval, HR=Heart rate, RR=Respiratory rate, Temp=Temperature, ABE=Base excess, Hct=Hematocrit, Na^+^=Sodium, K^+^=Potassium, Ca^2^+=Ionized calcium, Glu=Glucose, Lac=Lactate

## Discussion

This study investigated the comparison of electrocardiographic parameters of bovine neonates that experienced dystocia and eutocia. Until now, mobile ECGs have only been used sporadically, for example, to compare ECGs of calves of different ages or breeds [7, 18]. Dystocia commonly results in weakness and sudden death of calves, indicating that ECG abnormalities may be present in these animals.

The differences between laboratory diagnostic measures in the two groups could be attributed to differences in the conditions of birth. Calves born by dystocia suffer from hypercapnia and hypoxemia due to disturbed intrauterine fetomaternal gas exchange. Respiratory depression thus leads to respiratory acidosis. As the tissue does not receive sufficient oxygen, anaerobic glycolysis occurs, along with the increased formation of lactate. In this study, negative correlations between (1) respiratory rate and pH value and (2) respiratory rate and base deficit were observed in the dystocia group. This could be attributed to respiratory acidosis, which supports prior research that demonstrated the development of metabolic acidosis from respiratory acidosis [19]. The positive correlation between lactate levels and respiratory rate may also be explained by the body’s counter-regulation to tissue hypoxia [20]. Similarly, the increased concentration of ionized calcium in the dystocia group may be related to acidosis, as the accumulating H+ ions compete with ionized calcium for binding sites on protein molecules in the blood [21].

Clinical examination revealed a higher heart rate in calves of the dystocia group than the eutocia group over the entire study period of 5 days. This echoes Held *et al*. [22]’s results, in which tachycardia was described in neonates as a consequence of birth-related acidosis. Furthermore, studies involving sheep have demonstrated a direct correlation between oxygenation of the blood and heart rate [23]. To clarify whether the differences in heart rate between groups are due to oxygen saturation, arterial blood sampling should be performed. Due to the elevated level of care required for calves from dystocia, the increased heart rate may also be due to stress-induced activation of the autonomic sympathetic system [24]. In addition, myocardial damage due to birth-induced acidosis and hypoxia could cause a change in heartbeat frequency [25]. Thus, further research with respect to bovine cardiac biomarkers is needed.

Shortened P waves, QRS complexes, T waves, PQ intervals, and QT intervals were observed in calves from complicated births. The ST interval was longer in calves from births of dystocia than in those of the eutocia group. Amory *et al*. [7] detected that Belgian Blues had a significantly longer ST interval in comparison to Holstein Friesian calves. However, possible explanations for this were not discussed in the study. Regarding the results of our study, breeding differences seem likely. The prolonged ST interval could be due to the birth situation and the associated weakness due to hypoxia in the dystocia group. While the duration of the P wave and the QRS complex is not dependent on heart rate or physical stress, the shortened T waves, QT intervals, and PQ intervals may be explained by the increased heart rate of the calves [26–28]. Furthermore, Amory *et al*. [7] have demonstrated that beef cattle breeds have shortened ECG parameters, except for the aforementioned ST intervals. Differences in thoracic shapes, the thickness of the thoracic wall, cardiac mass, and the anatomical position and electrical axis of the heart within the thorax are postulated as possible causes for this. Of the 20 calves in each group, 15 in the dystocia group were beef cattle, whereas, in the spontaneous births group, only three of the 20 calves were beef cattle.

Of the ECG parameters studied, the QT interval and ST interval were found to be related to the course of birth. The other ECG parameters showed no interaction between group and time but differed significantly between the two groups. This could be due to the different breeds in the two groups. In human diabetes mellitus type 2 patients, prolonged QT intervals were observed during severe hypoglycemia [29]. Further changes in electrocardiographic parameters due to hypoglycemia are well-described in human medicine literature [30]. There were no significant group differences in the glucose level on the 1^st^ day of life in our study. Although no further laboratory measurements were performed, clinical follow-up examinations did not reveal any evidence of hypoglycemic disorders.

In addition, further correlations between ECG parameters and laboratory diagnostic values were observed in this study. In calves born by dystocia, the QT interval was negatively correlated to the lactate value but positively correlated to the potassium value. A shortening of the QT interval has already been described in the literature in the case of hyperkalemia [31]. Potassium is one of the essential electrolytes in cardiac cells. Related disorders, including hyper- and hypokalemia, may be life-threatening [32]. Disorders are primarily found in calves with neonatal diarrhea [31]. Therefore, this disease represents another potential avenue of study using the ECG. No significant differences were found in this study between groups regarding potassium. Unfortunately, no follow-up testing of laboratory parameters occurred, but it is conceivable that cellular damage due to acidosis or hypoxia could lead to an increase in potassium levels [33], and deviations in the ECG may arise as a result of this.

Finally, this study showed a negative correlation between the duration of the T wave and the internal body temperature in the eutocia group, while a negative correlation between the respiratory frequency and the QT interval was found in the dystocia group. The origin of these correlations should be investigated in more detail in further studies, as explanatory data do not yet exist in the literature.

## Limitations of the study

This study had some limitations. First, the breed distribution in the dystocia group may have skewed the results. Thus, whether observed differences are due to group membership (i.e., conditions of birth) or breed must be investigated in subsequent studies. Potential differences between dystocia and eutocia within dairy breeds (such as the Holstein Friesian, Jersey, and Brown Swiss) and beef cattle (such as the Charolais, Limousin, and Angus) should be explored in the future. In addition, research involving only one breed under different birth situations should also be conducted.

As venous blood samples were taken only within 24 h postpartum, further investigations are necessary to assess the potential correlations between laboratory measures and electrocardiographic parameters beyond the 1^st^ day of life. Arterial blood sampling could offer further insights into birth-induced acidosis and its influence on the 1^st^ days of life.

## Conclusion

This study is the first of its kind to observe that changes in ECG could be detected in calves born under conditions of dystocia. This factor could be associated with increased mortality in these animals. However, further studies should determine whether breed-specific deviations also play a role in this relationship.

## Data Availability

The data and the supporting tables that support the findings of this study are openly available in Giessen Electronic Library at http://geb.uni-giessen.de/geb/volltexte/2017/12403/.

## Authors’ Contributions

JN: Carried out the investigation. AW and MS: Designed the methodology. AW: Conceptualization, resources, and supervision. JJ: Drafted the original manuscript. All authors have read and approved the final manuscript.

## References

[1] Mee J.F (2013). Why do so many calves die on modern dairy farms and what can we do about calf welfare in the future?. Animals (Basel).

[2] Santman-Berends I.M.G, Schukken Y.H, van Schaik G (2019). Quantifying calf mortality on dairy farms:Challenges and solutions. J. Dairy Sci.

[3] Heringstad B, Chang Y.M, Svendsen M, Gianola D (2007). Genetic analysis of calving difficulty and stillbirth in Norwegian Red cows. J. Dairy Sci.

[4] Norquay R, Orr J, Norquay B, Ellis K.A, Mee J.F, Reeves A, Scholes S, Geraghty T (2020). Perinatal mortality in 23 beef herds in Orkney:Incidence, risk factors and aetiology. Vet. Rec.

[5] Hoedemaker M, Ruddat I, Teltscher M.K, Essmeyer K, Kreienbrock L (2010). Influence of animal, herd and management factors on perinatal mortality in dairy cattle--a survey in Thuringia, Germany. Berl. Munch. Tierarztl. Wochenschr.

[6] Berglund B, Steinbock L, Elvander M (2003). Causes of stillbirth and time of death in Swedish Holstein calves examined post mortem. Acta. Vet. Scand.

[7] Amory H, Rollin F.A, Genicot B.C, Beduin J.M, Lekeux P.M (1993). Comparative study of the body surface electrocardiogram in double-muscled and conventional calves. Can. J. Vet. Res.

[8] Matsui K, Sugano S, Masuyama I, Amada A, Kano Y (1984). Alterations in the heart rate of Thoroughbred horse, pony and Holstein cow through pre-and post-natal stages. Nihon Juigaku Zasshi.

[9] Brihoum M, Amory H, Desmecht D, Cassart D, Deleuze S, Rollin F (2010). Descriptive study of 32 cases of doxycycline-overdosed calves. J. Vet. Intern. Med.

[10] Weldon A.D, Moise N.S, Rebhun W.C (1992). Hyperkalemic atrial standstill in neonatal calf diarrhea. J. Vet. Intern. Med.

[11] Priyanka M, Mahendran K, Umapathi V, Dechamma H.J, Patel B.H.M, Reddy G.R, Sanyal A (2019). Successful treatment of cardiac dysrhythmia associated with foot and mouth disease in a calf. Iran. J. Vet. Res.

[12] Mahadappa P, Mahendran K, Winter R.L, Umapathi V, Krishnaswamy N, Gopalakrishnan A, Rao S, Gangaiah M, Kumar S, Patel B.H.M, Gautam N, Hegde R, Dechamma H.J, Sanyal A (2021). Characterization of arrhythmias, evaluation of cardiac biomarkers and their association with survival in calves suffering from foot-and-mouth disease. J. Vet. Cardiol.

[13] Akiyama N, Tagaino Y, Watanabe K.I, Horiuchi N, Kobayashi Y, Inokuma H (2021). A clinical case of single left ventricle in a Holstein calf. J. Vet. Med. Sci.

[14] Frese D.A, Thomason J.D, Reinhardt C, Bartle S, Rethorst D, Loneragan G.H, Thomson D (2017). Twenty-four hour Holter monitoring in finishing cattle housed outdoors. J. Vet. Cardiol.

[15] Chalmeh A, Karamifar S (2021). Evaluating heart electrical activities and cardiac arrhythmias of Holstein cows during ageing by short-term electrocardiography in comparison with 24-hour holter-monitoring. Vet. Med. Sci.

[16] Bonelli F, Vezzosi T, Meylan M, Nocera I, Ferrulli V, Buralli C, Meucci V, Tognetti R (2019). Comparison of smartphone-based and standard base-apex electrocardiography in healthy dairy cows. J. Vet. Intern. Med.

[17] Nagel C, Aurich J, Trenk L, Ille N, Drillich M, Pohl W, Aurich C (2016). Stress response and cardiac activity of term and preterm calves in the perinatal period. Theriogenology.

[18] Mendes L.C.N, Camacho A.A, Alves A.L.G, Borges A.S, Souza R.C.A, Ferreira W.L (2001). Standard electrocardiographic values in Holstein calves. Arq. Bras. Med. Vet. Zootec.

[19] Murray C.F, Leslie K.E (2013). Newborn calf vitality:Risk factors, characteristics, assessment, resulting outcomes and strategies for improvement. Vet. J.

[20] Wahl P, Bloch W, Mester J (2009). Moderne Betrachtungsweisen des Laktats:Laktat ein überschätztes und zugleich unterschätztes Molekül. Schweiz. Z. Sportmedizin.

[21] Sadiq N.M, Naganathan S, Badireddy M (2022). Hypercalcemia. StatPearls, Treasure Island, FL.

[22] Held T.H, Scheidegger A, Grunert E (1986). Cardiotocographic findings in healthy and asphyxiated cattle fetuses in the 2d stage of labor. Zentralbl. Veterinarmed. A.

[23] Bocking A.D (1993). The relationship between heart rate and asphyxia in the animal fetus. Clin. Invest. Med.

[24] Brunckhorst C.B, Holzmeister J, Scharf C, Binggeli C, Duru F (2003). Stress, depression and cardiac arrhythmias. Ther. Umsch.

[25] Daum S, Yang G.T (1986). Heart contractility in acute respiratory acidosis and acute hypoxia. J. Tongji Med. Univ.

[26] Busse M, Nißing A, Thomas M, Tegtbur U, Fikenzer S (2004). EKG-Parameter und Herzfrequenz bei Belastung:I. QT-Zeit und Herzfrequenz bei Belastung. Klinische Sportmedizin.

[27] Busse M, Nißing A, Thomas M, Tegtbur U, Fikenzer S (2004). EKG-Parameter und Herzfrequenz bei Belastung:V. QRS-Dauer und Herzfrequenz bei Belastung. Klinische Sportmedizin.

[28] Busse M, Nißing A, Thomas M, Tegtbur U, Fikenzer S, Miltzow S (2004). EKG-Parameter und Herzfrequenz bei Belastung:III. P-Dauer und Herzfrequenz bei Belastung. Klinische Sportmedizin.

[29] Hanefeld M, Ganz X, Nolte C (2014). Hypoglycemia and cardiac arrhythmia in patients with diabetes mellitus Type 2. Herz.

[30] Schäffer H, Bucka E, Friedländer K (1927). Über die Einwirkung des Insulins und der Hypoglykämie auf das menschliche Herz. Z. Ges. Exp. Med.

[31] Trefz F.M, Lorenz I, Constable P.D (2018). Electrocardiographic findings in 130 hospitalized neonatal calves with diarrhea and associated potassium balance disorders. J. Vet. Intern. Med.

[32] Teymouri N, Mesbah S, Navabian S.M.H, Shekouh D, Najafabadi M.M, Norouzkhani N, Poudineh M, Qadirifard M.S, Mehrtabar S, Deravi N (2022). ECG frequency changes in potassium disorders:A narrative review. Am. J. Cardiovasc. Dis.

[33] Andualem A.A, Yesuf K.A (2022). Incidence and associated factors of postoperative hypoxemia among adult elective surgical patients at Dessie comprehensive specialized hospital:An observational study. Ann. Med. Surg. (Lond.).

